# Transport of Nanoparticles and Tobramycin-loaded Liposomes in *Burkholderia cepacia* Complex Biofilms

**DOI:** 10.1371/journal.pone.0079220

**Published:** 2013-11-14

**Authors:** Anne-Sophie Messiaen, Katrien Forier, Hans Nelis, Kevin Braeckmans, Tom Coenye

**Affiliations:** 1 Laboratory of Pharmaceutical Microbiology, Ghent University, Ghent, Belgium; 2 Laboratory of General Biochemistry & Physical Pharmacy, Ghent University, Ghent, Belgium; 3 Center for Nano- and Biophotonics, Ghent University, Ghent, Belgium; The Scripps Research Institute and Sorrento Therapeutics, Inc., United States of America

## Abstract

Due to the intrinsic resistance of *Burkholderia cepacia* complex (Bcc) to many antibiotics and the production of a broad range of virulence factors, lung infections by these bacteria, primarily occurring in cystic fibrosis (CF) patients, are very difficult to treat. In addition, the ability of Bcc organisms to form biofilms contributes to their persistence in the CF lung. As Bcc infections are associated with poor clinical outcome, there is an urgent need for new effective therapies to treat these infections. In the present study, we investigated whether liposomal tobramycin displayed an increased anti-biofilm effect against Bcc bacteria compared to free tobramycin. Single particle tracking (SPT) was used to study the transport of positively and negatively charged nanospheres in Bcc biofilms as a model for the transport of liposomes. Negatively charged nanospheres became immobilized in close proximity of biofilm cell clusters, while positively charged nanospheres interacted with fiber-like structures, probably eDNA. Based on these data, encapsulation of tobramycin in negatively charged liposomes appeared promising for targeted drug delivery. However, the anti-biofilm effect of tobramycin encapsulated into neutral or anionic liposomes did not increase compared to that of free tobramycin. Probably, the fusion of the anionic liposomes with the negatively charged bacterial surface of Bcc bacteria was limited by electrostatic repulsive forces. The lack of a substantial anti-biofilm effect of tobramycin encapsulated in neutral liposomes could be further investigated by increasing the liposomal tobramycin concentration. However, this was hampered by the low encapsulation efficiency of tobramycin in these liposomes.

## Introduction

Cystic fibrosis (CF) is the most prevalent hereditary disease in the Caucasian population and is caused by mutations in both *cftr* alleles encoding a chloride channel [1]. The absence of a functional chloride channel (CFTR channel) results in the secretion of thick, viscous mucus in several organs, including the lungs and the gastrointestinal tract [2]. The presence of thick mucus in the lungs impairs the mucociliary clearance, rendering CF patients more susceptible to lung infections. These lung infections are not sufficiently cleared and rapidly evolve in chronic infections, the main cause of morbidity and mortality in CF patients [3]. The most significant pathogen colonizing the CF lungs is *Pseudomonas aeruginosa*. Over time, approximately 80% of the CF population becomes infected with this pathogen [4]. Compared to *P. aeruginosa*, *Burkholderia cepacia* complex (Bcc) infections only account for a small percentage of the respiratory infections within the CF population. However, they are often associated with rapid deterioration of the lung function and increased mortality [5]. The capacity of Bcc species to cause invasive disease and their high level of intrinsic antibiotic resistance make them particularly difficult to eradicate. Through the production of different exopolymeric substances (EPS) additional protection is provided against antibiotics and host immune components [6]. As there is still no optimal treatment regimen for Bcc infections in CF patients [7], these pathogens continue to be highly problematic [8] emphasizing the urgent need for effective antibiotic therapies. As for *P. aeruginosa*, the production of EPS by Bcc strains is essential for the formation of thick and mature biofilms [9]. These EPS, including polysaccharides and extracellular DNA (eDNA), can delay the penetration of an antibiotic through the biofilm [10]. By the encapsulation of the antibiotic in a liposomal carrier, interactions between the antibiotic and EPS could be avoided, resulting in an improved anti-biofilm effect. In addition, liposome-encapsulated antibiotics are protected from degradation by antibiotic-inactivating enzymes (like β-lactamases) which can accumulate in the biofilm matrix [11]. Another important factor contributing to antibiotic resistance in several Gram-negative bacteria, including *P. aeruginosa* and Bcc species, is their low outer membrane permeability [12]–[14]. The uptake of antibiotics encapsulated in neutral liposomes is not affected by this outer membrane barrier as this can occur through direct fusion of the liposome with the bacterial membrane [15]. It has been shown that subinhibitory concentrations of tobramycin encapsulated in neutral liposomes displays a high bactericidal activity against planktonic cultures of several bacterial species, including *P. aeruginosa* and *B. cenocepacia* [16]–[18], probably due to an enhanced uptake of the antibiotic. Besides the improved transport through the biofilm and a better uptake in the cell, liposomes could provide selective delivery to the target site by incorporating specific ligands to the liposome surface. Currently, several liposome based drugs have been approved for clinical use and various others are in clinical trials [19]. Arikace, a neutral liposomal amikacin formulation, currently undergoes phase III clinical trials for the treatment of lung infections [19], [20]. It is the first liposomal drug being investigated for aerosol delivery.

In the present study, we investigated whether the bactericidal effect of tobramycin against Bcc biofilms could be increased by encapsulating the antibiotic in liposomes. Therefore, we analyzed the behavior of positively and negatively charged nanoparticles in Bcc biofilms by means of single particle tracking (SPT) as a model for liposomal transport. According to our observations, negatively charged particles were directed towards cell clusters, while positively charged particles became immobilized by interactions with fiber-like structures (likely eDNA) in the biofilm matrix. Based on these results, tobramycin encapsulated in anionic liposomes could be promising in terms of targeted drug delivery. We consequently evaluated the activity of neutral (reference) and anionic liposomal tobramycin formulations against Bcc biofilms.

## Materials and Methods

### Strains

We used 5 Bcc strains in the present study: *B. cenocepacia* LMG 16656 and LMG 18829, *B. cepacia* LMG 1222, *B. multivorans* LMG 18825 and *B. dolosa* LMG 18943. All Bcc strains were obtained from the BCCM/LMG Bacteria Collection (Ghent, Belgium) or were kindly provided by Dr. P. Vandamme (Ghent University, Belgium). The bacteria were stored in Microbank tubes (Prolab Diagnostics, Richmond Hill, ON, Canada) at −80°C and transferred twice on Mueller Hinton (MH) (Lab M, Heywood, UK) agar plates before use in any experiment.

### Lipids

1,2-dipalmitoyl-sn-glycero-3-phosphocholine (DPPC) and cholesterol were obtained from Sigma-Aldrich (St. Louis, MO, USA) and stored at −20°C. 1,2-dioleoyl-sn-glycero-3-phosphocholine (DOPC) and 1,2-dipalmitoyl-sn-glycero-3-phosphoglycerol sodium salt (DPPG), were obtained from Corden Pharma International (Plankstadt, Germany) and stored at −20°C. Lipids were dissolved in chloroform and stock solutions were stored between 2–8°C.

### Nanoparticles

Yellow-green (λ_ex_:505/λ_em_:515) fluorescent carboxylate-modified polystyrene FluoSpheres of 0.2 µm diameter were purchased from Invitrogen (Carlsbad, CA, USA). Positively charged nanospheres were prepared from the carboxylate-modified FluoSpheres by modification with N,N-dimethylethylenediamine (DMEDA) (Sigma-Aldrich) as described previously [21]. The size and zeta-potential of the positively charged nanospheres were measured in HEPES (pH 7.3, 20 mM) using the Zetasizer Nano-ZS (Malvern, Worcestershire, UK).

### Single Particle Tracking Setup

All SPT measurements were performed on a custom-built laser wide-field fluorescence microscope setup as described previously [22]. Briefly, two solid-state lasers, a 100 mW Calypso 491 nm (Cobolt, Solna, Sweden) and a 100 mW Spectra Physics Excelsior 642 nm (CA, USA), were used to excite the fluorophores in samples mounted on a TE2000-E (Nikon BeLux, Brussels, Belgium) inverted microscope equipped with a Plan Apo VC 100×1.4 NA oil immersion objective lens (Nikon). A diffuser in the illumination path ensured even illumination of the sample. An acousto-optical tunable filter (AOTF) was used to modulate the intensity of the laser beams. The AOTF was synchronized to the EMCCD camera (Cascade II:512; Roper Scientific, Tucson, AZ, USA) in order to limit photobleaching during imaging. Videos of the moving nanoparticles were acquired with the NIS Elements software package (Nikon).

### Analysis of SPT Data

Analysis of the videos was performed off-line using software developed by Braeckmans et al. [23]. First, the particles were localized in all frames of the SPT movie. A selection of “real” particles was made according to user-defined criteria, including size, contrast relative to the local background and sphericity. Subsequently, individual trajectories of the nanospheres were calculated using a nearest neighbor algorithm. The positions of nanospheres that are closest to each other were connected in subsequent image frames, taking into consideration the maximum distance a particle can reasonably move from one frame to another. The trajectories were further analyzed based on mean square displacement (*MSD*) analysis. The MSD was calculated for every available time lag (*t*), i.e. the multiples of time between two subsequent images in an SPT movie.

A weighted least squares fitting was performed of
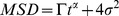
to the *MSD* vs. *t* curves, yielding for each trajectory the parameters Gamma (*Γ*), alpha (α) and sigma (*σ*). Gamma is the so-called transport coefficient, alpha the anomalous exponent and sigma a parameter taking into account the limited precision with which a particle can be localized. The parameter of interest was alpha, which contains information on the mode of motion. For free diffusion, *α* equals 1 while *α* <1 and *α* >1 represent sub- and super-diffusion, respectively. By analyzing the *MSD* versus *t* plots of many trajectories, a distribution of *α* values was obtained. In addition, the apparent diffusion coefficient, *D_a_*, was calculated corresponding to the first time lag according to:



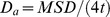



The distribution of *D_a_* values was further processed using a maximum entropy method (MEM) which improves the resolution and at the same time smoothes the curve by only retaining the features that are statistically warranted by the data [22].

### SPT Measurements in Biofilms

Bcc biofilms were cultured in uncoated 35 mm glass bottom culture dishes (MatTek, Ashland, MA, USA). The dishes were inoculated with 2 mL of a standardized bacterial suspension (∼10^8^ CFU/mL in MH broth) in the presence or absence of 10 µg/mL dornase alfa (Pulmozyme, Genentech, SF, USA) and were incubated at 37°C. After 4 h, the supernatant, containing non-adhered cells, was removed and adhered cells were carefully washed with physiological saline (0.9% w/v NaCl) (PS). Subsequently, 2 mL of fresh MH broth (with or without 10 µg/mL dornase alfa) was added and the dishes were incubated for another 20 h at 37°C. After 24 h of biofilm formation, the supernatant was removed and biofilms were gently washed with PS. Biofilm cells were stained by adding 1 mL of a Syto 59 (Invitrogen) solution (5 mM in PS). After 15 min of incubation at room temperature, protected from light, the biofilm was washed twice with PS. The nanoparticle stock suspensions were sonicated for 10 min before dilution in PS (∼0.002% solids). One mL of this particle suspension was added to the biofilms, right before recording movies. Movies of 5 s with a temporal resolution of 39.2 ms (time between two subsequent images, time lag) and an image acquisition time of 3 ms were recorded. Dual color image acquisition allowed easy navigation within the biofilm. Between 25 and 30 movies on different locations in the biofilm were recorded. Each experiment was carried out in triplicate at room temperature.

### Liposome Preparation

Liposomes were prepared by a dehydration-rehydration method as described previously [24]. In brief, for the preparation of neutral liposomes, 50 µmol of DPPC and 25 µmol of cholesterol were dissolved in 1 mL of chloroform in a round-bottomed flask. For the anionic liposomes, 53 µmol of DOPC and 6.6 µmol of DPPG were dissolved in 1 mL of chloroform. The round-bottomed flask was connected to a rotary evaporator to dry the lipid film under controlled vacuum at 50°C. The lipid film was rehydrated with 2 mL of a sucrose solution in distilled water (1∶1, w/w, sucrose to lipid). Sucrose is needed to stabilize the liposomes during freeze drying. Lipid suspensions were vortexed to form multilamellar vesicles and then sonicated for 5 min in an ultrasonic bath (Branson 3510), followed by two additional cycles of vortexing and sonication. The resulting suspension was then mixed with 1 mL (40 mg/mL) of tobramycin in PS. The mixture was frozen (−20°C) and immediately freeze dried. After freeze drying, the powdered formulations were stored at 4°C until use. For rehydration, 200 µL of distilled water at 50°C was added. The suspension was vortexed and incubated for 30 min at 50°C. These steps were repeated with 200 µL phosphate-buffered saline (PBS, pH 5.9). After incubation at 50°C, 1.6 mL of PBS was added, the mixture was vortexed and incubated for another 30 min at 50°C. Non-encapsulated tobramycin was removed following three rounds of PBS wash (18300×g, 15 min at 4°C) and the pellet was resuspended in 2 mL PS. The size and zeta-potential of the liposomes were measured in HEPES (pH 7.3, 20 mM) using the Zetasizer Nano-ZS (Malvern, Worcestershire, UK).

### Quantification of the Amount of Encapsulated Tobramycin

The concentration of liposome encapsulated tobramycin was measured by agar diffusion, using a laboratory strain of *Bacillus subtilis* (ATCC 6633) as indicator organism. *B. subtilis* ATCC 6633 spore suspensions were prepared as described in the European Pharmacopoeia [25]. In brief, *B. subtilis* was grown at 35–37°C for 7 days on Antibiotic medium 1 (AM1) supplemented with 0.001 g/L manganese sulphate. After at least 7 days, the growth, which mainly consisted of spores, was washed off using sterile water. The obtained suspension was heated at 70°C for 30 min and diluted to give an appropriate concentration of spores (10^7^–10^8^ per mL). The spore suspensions were stored at 4°C until use. We used this suspension to prepare a 1% suspension of spores (4 mL/400 mL) in warm (47.5°C) AM 11 agar. The agar was poured into a sterile glass plate and was left to solidify for 15 min at room temperature. To lyse the liposomes, 0.2% Triton X-100 was added to the liposomal solutions. This level of Triton X-100 had no effect on the performance of the assay (data not shown). Wells of 5 mm diameter were punched in the agar and filled with 200 µL of standard tobramycin solutions or with the sample. The plate was covered with a steel lid and incubated for 20 hours at 35°C after 4 h of pre-diffusion at 4°C. We subsequently measured the inhibition zones and the average of 4 measurements was used for data analysis. A standard curve of known concentrations of free tobramycin (0.03125–1 µg/mL) was constructed and was utilized to calculate the amount of encapsulated tobramycin that was released after Triton X-100 treatment.

### Determination of the MIC of Tobramycin

MICs were determined in duplicate according to the EUCAST broth microdilution protocol using unsupplemented MH broth. In brief, bacterial suspensions were standardized at ∼5 × 10^5^ CFU/mL before inoculation of a flat-bottomed 96-well plate filled with 100 µL of tobramycin serial dilutions in MH. The range of tobramycin concentrations used was from 2 to 1024 mg/L. Plates were incubated at 37°C for 20 h and the optical density was determined at 590 nm using a multilabel microtitre plate reader (Envision, Perkin Elmer LAS, Waltham, MA, USA). The MIC is the lowest antibiotic concentration for which a similar optical density was observed in the inoculated and blank wells.

### Activity of Liposome Encapsulated Tobramycin Against Bcc Biofilms

Biofilms were cultured in round-bottomed 96-well plates. An inoculum was prepared by suspending bacteria from a pure culture on MH agar in MH broth. The inoculum was standardized at ∼10^8^ CFU/mL and the wells of the microtitre plate were filled with 100 µL of this suspension. Plates were incubated at 37°C without shaking. After 4 h, the supernatant, containing planktonic cells, was aspirated from the wells. Adhered cells were carefully washed with 100 µL of PS and 100 µL of fresh sterile MH broth was added. Plates were incubated for an additional 20 h at 37°C. After 24 h of biofilm formation, biofilms were washed with 100 µL of PS before treatment with free tobramycin (final concentrations of 140 µg/mL or 4XMIC of tobramycin in PS) or liposome encapsulated tobramycin (final concentrations of 140 µg/mL or 4XMIC of tobramycin in PS). After 24 h of treatment at 37°C, cell numbers were determined by plate counting.

## Results

### Properties of Nanoparticles and Liposomes

The average sizes and average zeta-potentials of nanospheres and liposomes used in the present study are shown in Table 1. For the liposomes, the concentrations of encapsulated tobramycin (i.e. the concentration that is released after breaking up all liposomes in the solution with Triton X-100) are also shown in Table 1.

**Table 1:** Characteristics of the nanospheres and liposomes used in the present study (n = 3).

Particle	Average size(nm) (±SD)	Average zeta-potential(mV) (±SD)	Total lipsomal tobramycin concentration(µg/mL) (±SD)
Carboxylate-modified nanosphere	224.0 (±0.7)	−48.4 (±0.7)	/
DMEDA-modified nanosphere	231.9 (±1.2)	30.4 (±0.6)	/
DPPC/cholesterol liposomes(2/1, molar ratio)	426.3 (±26.4)	−0.5 (±0.1)	141 (±35)
DOPC/DPPG liposomes(8/1, molar ratio)	228.5 (±34.9)	−22.3 (±0.5)	1128(±16)

### Transport of Negatively Charged Nanoparticles in Bcc Biofilms

**Figure 1:**
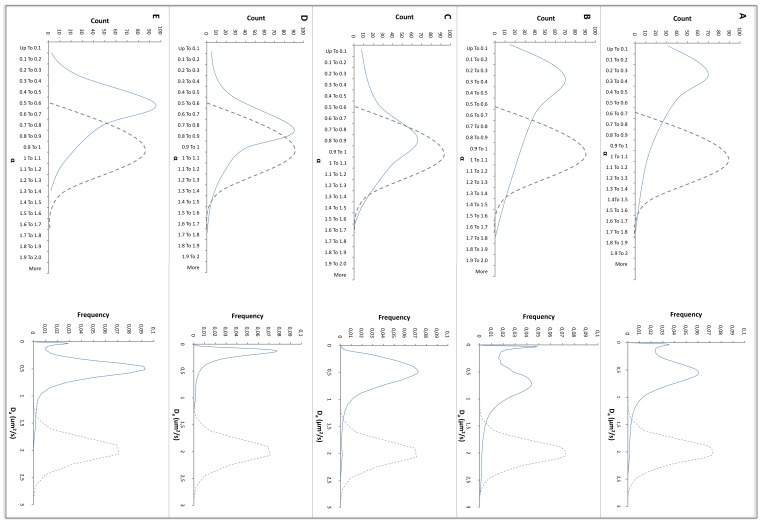
Mobility of 0.2 µm carboxylate-modified nanospheres in Bcc biofilms.

The mobility of carboxylate-modified polystyrene nanospheres (0.2 µm) added to Bcc biofilms was studied by analyzing individual trajectories of the nanospheres in the biofilm. The distribution of *α* values and *D_a_* coefficients of the carboxylate-modified nanospheres is shown in Figure 1. Average *α* and *D_a_* values for all conditions are shown in Table 2. For all strains investigated, negatively charged particles display subdiffusion (0<*α*<1 ) when added to the biofilm. This most pronounced subdiffusion is observed in *B. cenocepacia* LMG 16656 and *B. cenocepacia* LMG 18829 biofilms and to a lesser extent in *B. multivorans* LMG 18825 and *B. cepacia* LMG 1222 biofilms. This is also reflected in the *D_a_* distributions where an immobilized fraction is observed in both *B. cenocepacia* LMG 16656 and *B. cenocepacia* LMG 18829 biofilms. This anomalous diffusion indicates that the negatively charged particles strongly interact with the biofilms. In addition, the particles diffuse slowly in the biofilm as their apparent diffusion coefficients are much lower than those in water (*D_w_* = 1.96 µm^2^/s (26)). The highest diffusion rates were observed in a *B. cepacia* LMG 1222 biofilm (average *D_a_* = 0.42 µm^2^/s) while the lowest were observed in a *B. multivorans* LMG 18825 biofilm (average *D_a_* = 0.15 µm^2^/s). The mobility of the negatively charged nanospheres in both *B. cenocepacia* LMG 16656 and LMG 18829 biofilms is similar, yielding average diffusion coefficients of 0.26 and 0.28, respectively.

**Table 2:** Average apparent diffusion coefficients, both in Bcc biofilms and in water [26], and average α values of the nanospheres in Bcc biofilms.

Biofilm	Condition	Particle	Av Da (µm^2^/s) (±SD)	Av α	Av Dw (µm^2^/s) (±SD)
*B. cenocepacia* LMG 16656	Control	Carboxylate-modified	0.26 (±0.29)	0.43	1.96 (±0.02)
	Control	DMEDA-modified	0.29 (±0.21)	0.39	1.88 (±0.09)
	Dornase α	DMEDA-modified	0.37 (±0.44)	0.44	
*B. cenocepacia* LMG 18829	Control	Carboxylate-modified	0.28 (±0.36)	0.48	1.96 (±0.02)
	Control	DMEDA-modified	0.23 (±0.30)	0.23	1.88 (±0.09)
	Dornase α	DMEDA-modified	0.54 (±0.30)	0.49	
*B. cepacia* LMG 1222	Control	Carboxylate-modified	0.42 (±0.25)	0.81	1.96 (±0.02)
	Control	DMEDA-modified	0.27 (±0.28)	0.62	1.88 (±0.09)
	Dornase α	DMEDA-modified	0.45 (±0.26)	0.97	
*B. multivorans* LMG 18825	Control	Carboxylate-modified	0.15 (±0.11)	0.77	1.96 (±0.02)
	Control	DMEDA-modified	0.38 (±0.20)	0.65	1.88 (±0.09)
	Dornase α	DMEDA-modified	0.42 (±0.30)	0.67	
*B. dolosa* LMG 18943	Control	Carboxylate-modified	0.32 (±0.36)	0.57	1.96 (±0.02)
	Control	DMEDA-modified	0.25 (±0.30)	0.35	1.88 (±0.09)
	Dornase α	DMEDA-modified	0.19 (±0.30)	0.53	

### Transport of Positively Charged Nanoparticles in Bcc Biofilms

**Figure 2:**
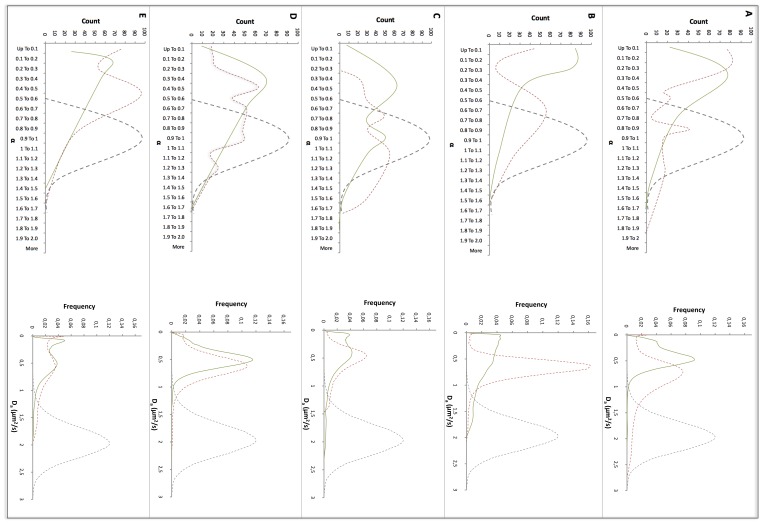
Mobility of 0.2 µm DMEDA- modified nanospheres in Bcc biofilms.

The mobility of DMEDA-modified polystyrene nanospheres (0.2 µm) in different Bcc biofilms was also studied (Figure 2). Positively charged nanospheres displayed subdiffusion in all Bcc biofilms grown in the absence of dornase alfa. Alfa values for *B. cepacia* LMG 1222 show a bimodal distribution, with part of the particles displaying free diffusion. Anomalous subdiffusion was most pronounced in *B. cenocepacia* LMG 18829 (mean *α* = 0.23). In contrast, *α* values closest to 1 were obtained in *B. multivorans* LMG 18825 and *B. cepacia* LMG 1222 biofilms, with mean α values of 0.65 and 0.62, respectively. This correlates with the highest average apparent diffusion coefficient (average *D_a_* = 0.44) in *B. multivorans* LMG 18825 biofilms. Although the average diffusion coefficient in *B. cepacia* LMG 1222 is much smaller, the distribution is clearly bimodal, with two groups of particles behaving differently. The peak of the faster moving population represents particles with an apparent diffusion coefficient of 0.41 µm^2^/s. Still, positively charged nanospheres diffuse much more slowly in the Bcc biofilms than in water (*D_w_* = 1.88 µm^2^/s [26]). The hindered transport of positively charged particles in Bcc biofilms could be due to interaction with negatively charged matrix components. It was previously observed that cationic nanospheres interacted with fiber-like structures in Bcc biofilms, greatly impairing their mobility and this was confirmed in the present study (Movie S1 and Movie S2). As these fiber-like structures are likely eDNA, the experiments with DMEDA-modified nanospheres were repeated in biofilms grown in the presence of dornase alfa (10 µg/mL) (Figure 2). The most pronounced effect of dornase alfa on the mobility of positively charged nanospheres was observed in *B. cepacia* LMG 1222 and *B. cenocepacia* LMG 18829 biofilms for which average diffusion coefficients approximately doubled, from 0.27 to 0.45 µm^2^/s and from 0.23 to 0.54 µm^2^/s, respectively. In addition the α distributions of the positively charged nanospheres displayed a shift to higher values, although this shift was less pronounced in *B. cenocepacia* LMG 16656 and *B. multivorans* LMG 18825 (Figure 2).

### Activity of Liposome Encapsulated Tobramycin Against Bcc Biofilms

**Figure 3:**
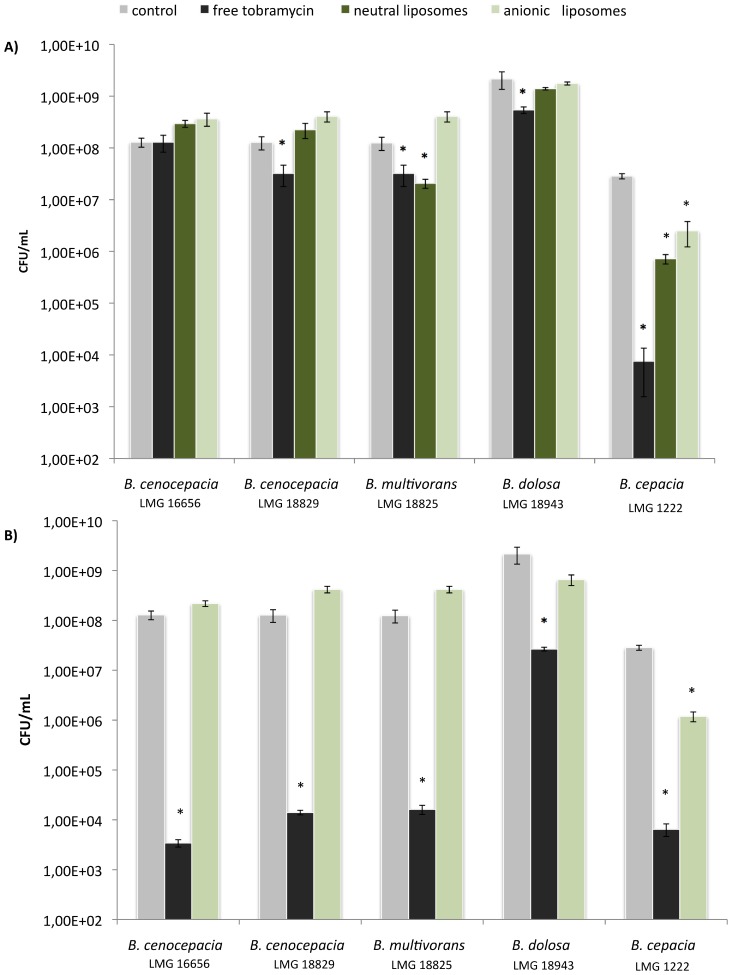
Activity of free and liposomal tobramycin against Bcc biofilms.

We tested the activity of both neutral and negatively charged liposomes containing tobramycin against Bcc biofilms (Figure 3). When added at a final concentration of 140 µg/mL (Figure 3a), free tobramycin showed modest activity against *B. cenocepacia* LMG 18829, *B. multivorans* LMG 18825 and *B. dolosa* LMG 18943 and strong activity against *B. cepacia* LMG 1222. This is in agreement with the MIC values observed. The MIC for *B. cepacia* LMG 1222 is low (32 µg/mL) while these for *B. cenocepacia* LMG 18829, *B. multivorans* LMG 18825 and *B. dolosa* LMG 18943 were only slightly below (128 µg/mL) the tobramycin concentration tested (140 µg/mL). Finally, the MIC of tobramycin for *B. cenocepacia* LMG 16656 (256 µg/mL) is considerably above the concentration used in this experiment, explaining the lack of bactericidal activity (Figure 3A). Both neutral and negatively charged liposomes containing 140 µg/mL of tobramycin (≈ 4XMIC) showed a bactericidal effect against *B. cepacia* LMG 1222 biofilms but no increased bactericidal activity compared to free tobramycin was observed. In *B. multivorans* LMG 18825 biofilms, neutral liposomes containing 140 µg/mL tobramycin (≈ MIC) showed the same activity as free tobramycin. Previous research from our group has indicated that tobramycin at concentrations of 4XMIC yielded a substantial bactericidal effect against Bcc biofilms [27]. Increasing the liposomal tobramycin concentration to 4XMIC was only possible for the negatively charged liposomes as the maximum achievable concentration of encapsulated tobramycin in neutral liposomes was 140 µg/mL. However, at this increased tobramycin concentration, negatively charged liposomal tobramycin formulations only showed bactericidal activity against *B. cepacia* LMG 1222 (Figure 3B).

## Discussion

In the present study we investigated the transport of negatively and positively charged nanospheres in Bcc biofilms. The transport of nanospheres can be used as a model to predict the transport of antibiotic-containing liposomes. Liposomes are made up of phospholipids and can act as biodegradable delivery systems. Antibiotics encapsulated in liposomes have lower toxicity and higher bioactivity and bioavailability [18], [28]. It has already been demonstrated that liposomal antibiotic formulations show increased bactericidal activity against biofilms compared to free antibiotics [29], [30]. However, a first condition is that efficient transport of liposomes in the biofilm matrix takes place. Therefore, as a first step in developing a drug-delivery system with activity against Bcc biofilms, we studied the transport of model nanospheres in these biofilms. In a previous study we have shown that PEGylated neutral nanospheres displayed normal (unobstructed) diffusion in *B. multivorans* LMG 18825 biofilms and that these particles diffuse in the biofilm at similar rates as in water [26]. This was in contrast to the mobility of carboxylate- and DMEDA-modified nanopsheres in a *B. multivorans* LMG 18825 biofilm which was found to be strongly impeded [26]. In the present work we expanded the panel of Bcc strains to study the diffusion in biofilms of both carboxylate- and DMEDA-modified nanospheres. The negatively charged nanospheres moved 5 to 13 times more slowly in all Bcc biofilms tested compared to in water. In all biofilms, a fraction of relatively immobile negatively charged particles, characterized by *D_a_* values close to zero, was observed. This immobilization is probably caused by electrostatic interactions between positively charged components of the biofilm and the negatively charged nanospheres, which correlates with the average *α* <1 values observed for these particles, indicating anomalous subdiffusion. It was previously observed that positively charged particles are immobilized on fiber-like structures, which were hypothesized to be eDNA [26]. Here we investigated this further by studying the transport of DMEDA-modified nanospheres in biofilms grown in the presence of dornase alfa. In general it was found that dornase alfa treatment reduced the extent of anomalous diffusion and improved the diffusion rate, supporting the view that positively charged particles can get trapped by binding to eDNA in the biofilm matrix. As DMEDA-modified nanospheres interact with eDNA, the application of positively charged liposomes as antibiotic carrier systems can only be considered in combination with e.g. dornase alfa. However, as previous research has indicated that the use of dornase alfa could be contraindicated in CF patients infected with *B. cenocepacia* [31], we did not test the effect of tobramycin encapsulated in positively charged liposomes. Instead, the effect of tobramycin encapsulated in negatively charged liposomes was compared to that of tobramycin encapsulated in neutral liposomes. Neutral liposomal formulations were tested as a reference as these appear, at first sight, less interesting to treat Bcc biofilm infections based on the mobility of neutral nanospheres which showed no enrichment in *B. multivorans* LMG 18825 biofilms. In contrast, enrichement of carboxylate-modified nanospheres at sites close to the bacteria has been observed [26]. Therefore, if anionic liposomes behave similarly in Bcc biofilms as anionic nanospheres, tobramycin could potentially be targeted near the cell clusters when encapsulated in anionic liposomes, resulting in an increased anti-biofilm effect. At free tobramycin concentrations equal to 4XMIC, a substantial bactericidal effect against all Bcc biofilms was observed. Unfortunately, this anti-biofilm effect could not be increased by encapsulating tobramycin in either neutral or anionic liposomes. As the surface of Bcc cells is negatively charged it could be that the fusion of anionic liposomes with the bacterial cells is limited by repulsive forces at close proximity. The bactericidal activity of tobramycin encapsulated in neutral liposomes at concentrations of 4XMIC could only be tested against *B. cepacia* LMG 1222 as this strain showed the lowest MIC (32 µg/mL) and not more than 140 µg/mL of tobramycin could be encapsulated in these neutral liposomes. The neutral liposomal tobramycin formulation did not show an increased bactericidal effect compared to that of free tobramycin. Although the observed relatively unobstructed motion of neutral particles in *B. multivorans* LMG 18825 biofilms suggests no penetration difficulties for neutral liposomes in the biofilm, we did not observe a substantial anti-biofilm effect. However, the effect of the neutral liposomal tobramycin at a concentration of 140 µg/mL was not decreased compared to the effect of free tobramycin in *B. multivorans* LMG 18825 biofilms, indicating that the encapsulated tobramycin does enter the cell. Possible explanations for the lack of an anti-biofilm effect of neutral formulations of liposomal tobramycin in *B. cenocepacia* LMG 16656, *B. cenocepacia* LMG 18829 and *B. dolosa* LMG 18943 are that a hydrophilic EPS layer in close proximity to the biofilm cells protects them from fusion with the liposomal carrier system. Alternatively, it could be that the transport of nanospheres is not a good model for the transport of liposomes in Bcc biofilms, especially for the neutral liposomes which are twice the size of the nanospheres. Smaller sized neutral liposomes with equal tobramycin encapsulation efficiency would allow to investigate this further but the low encapsulation efficiency of tobramycin into neutral liposomes [24] makes this impossible at present.

## Conclusions

Both positively- and negatively charged nanospheres show slow subdiffusion in Bcc biofilms. While positively charged nanospheres likely interact with eDNA, negatively charged nanospheres probably bind to positively charged matrix components. As previous research from a collaborating research group has indicated an enrichment of negatively charged nanospheres close to biofilm clusters, anionic liposomes could possibly serve as antibiotic delivery systems with high potential for treatment of Bcc biofilm infections. However, anionic liposomal formulations with tobramycin concentrations of 4XMIC only displayed bactericidal activity against *B. cepacia* LMG 1222 biofilms. In contrast, free tobramycin at this concentration showed high bactericidal activity against all Bcc biofilms tested. The fusion of the negatively charged liposome with the negatively charged bacterial membrane is likely limited by electrostatic repulsive forces at close proximity. Although we observed unobstructed diffusion of neutral nanospheres, the use of a neutral liposomal tobramycin formulation did not result in an increased anti-biofilm effect compared to free tobramycin. Additional research for the development of an optimal tobramycin carrier to treat Bcc biofilm infections is needed.

## Supporting Information

MovieS1 **Movement of cationic particles in** ***B. cenocepacia*** **LMG 16656 biofilm.**
(AVI)

MovieS2 **Movement of cationic particles in** ***B. dolosa*** **LMG 18943 biofilm.**
(AVI)
